# Proteomic Profile of Mouse Brain Aging Contributions to Mitochondrial Dysfunction, DNA Oxidative Damage, Loss of Neurotrophic Factor, and Synaptic and Ribosomal Proteins

**DOI:** 10.1155/2020/5408452

**Published:** 2020-06-09

**Authors:** Yingchao Li, Haitao Yu, Chongyang Chen, Shupeng Li, Zaijun Zhang, Hua Xu, Feiqi Zhu, Jianjun Liu, Peter S. Spencer, Zhongliang Dai, Xifei Yang

**Affiliations:** ^1^Key Laboratory of Modern Toxicology of Shenzhen, Shenzhen Center for Disease Control and Prevention, Shenzhen 518055, China; ^2^College of Pharmacy, Jinan University, Guangzhou 510632, China; ^3^State Key Laboratory of Oncogenomics, School of Chemical Biology and Biotechnology, Peking University Shenzhen Graduate School, Shenzhen 518055, China; ^4^Institute of New Drug Research and Guangzhou, Key Laboratory of Innovative Chemical Drug Research in Cardio-Cerebrovascular Diseases, Jinan University College of Pharmacy, Guangzhou 510632, China; ^5^Cognitive Impairment Ward of Neurology Department, The 3rd Affiliated Hospital of Shenzhen University, China; ^6^Department of Neurology, School of Medicine, Oregon Institute of Occupational Health Sciences, Oregon Health & Science University, Portland, Oregon 97239, USA; ^7^The Department of Anesthesiology, Shenzhen People's Hospital, The Second Clinical Medical College, Jinan University, Shenzhen 518020, China

## Abstract

The deleterious effects of aging on the brain remain to be fully elucidated. In the present study, proteomic changes of young (4-month) and aged (16-month) B6129SF2/J male mouse hippocampus and cerebral cortex were investigated by using nano liquid chromatography tandem mass spectrometry (NanoLC-ESI-MS/MS) combined with tandem mass tag (TMT) labeling technology. Compared with the young animals, 390 hippocampal proteins (121 increased and 269 decreased) and 258 cortical proteins (149 increased and 109 decreased) changed significantly in the aged mouse. Bioinformatic analysis indicated that these proteins are mainly involved in mitochondrial functions (FIS1, DRP1), oxidative stress (PRDX6, GSTP1, and GSTM1), synapses (SYT12, GLUR2), ribosome (RPL4, RPS3), cytoskeletal integrity, transcriptional regulation, and GTPase function. The mitochondrial fission-related proteins FIS1 and DRP1 were significantly increased in the hippocampus and cerebral cortex of the aged mice. Further results in the hippocampus showed that ATP content was significantly reduced in aged mice. A neurotrophin brain-derived neurotrophic factor (BNDF), a protein closely related with synaptic plasticity and memory, was also significantly decreased in the hippocampus of the aged mice, with the tendency of synaptic protein markers including complexin-2, synaptophysin, GLUR2, PSD95, NMDAR2A, and NMDAR1. More interestingly, 8-hydroxydeoxyguanosine (8-OHdG), a marker of DNA oxidative damage, increased as shown by immunofluorescence staining. In summary, we demonstrated that aging is associated with systemic changes involving mitochondrial dysfunction, energy reduction, oxidative stress, loss of neurotrophic factor, synaptic proteins, and ribosomal proteins, as well as molecular deficits involved in various physiological/pathological processes.

## 1. Introduction

Molecular and cellular changes occurring with the passage of time provide an indispensable foundation with which to detect and define deviations from the normal aging process that surfaces in the form of various neurodegenerative diseases [1, 2]. While a minority of these diseases results from defined genetic mutations that can be modeled in transgenic rodents, especially mice, many occur sporadically and develop with aging. Therefore, understanding the evolution of brain aging would be of paramount importance to provide novel molecular and cellular clues leading to neurodegenerative diseases. To determine the specific molecular mechanisms and related biomarkers of brain aging, we have used 4- and 16-month-old B6129SF2/J mice to study the systemic changes of proteins in the hippocampus and cortex by using nano liquid chromatography tandem mass spectrometry (NanoLC-ESI-MS/MS) coupled with tandem mass tag (TMT) labeling technology, a robust, sensitive, and accurate high-resolution analytical method [3].

Several studies of physiological brain aging in mice have been published, some of which have examined gene expression changes and, more recently, proteomic differences. These studies have employed mice of various strains (BALB/c, C57BL/6NHsd, and C57BL/10J), ages (range of 1-30 months), and sexes (males, females, or both), with comparisons variably among various brain areas of the cerebral cortex, hypothalamus, and cerebellum [4–10]. A recent quantitative proteomic analysis of the hippocampus, cortex, and cerebellum of postnatal (1 month) and middle-aged (12 months) C57BL/10J mice found total protein expression levels to be similar in the two age groups, and the hippocampus showed the most variable in protein expression across age [10]. The ability of aging neurons to oxidize glucose through glycolysis and mitochondria, as well as the ability to utilize fatty acids, increases and decreases from early to middle life (12 months) [9]. However, till now, the general picture of systemic molecular changes with aging, which was proposed to involve metabolic, immunological, inflammatory, and cellular functional, has now been explored. In the present study, hippocampal and cerebral cortical quantitative proteomics were explored through the age of 16 months (relative to 4 months) in a related mouse strain (B6129SF2/J). Our results showed that aging accompanying protein changes are related to mitochondrial dynamics, energy metabolism, GTPase function, oxidative stress, ribosome, synapses, loss of neurotrophic factor, and transcriptional regulation, among others.

## 2. Materials and Methods

### 2.1. Animals and Treatment Protocol

Animal treatment and housing were carried out in accordance with the Principles of Laboratory Animal Care (NIH publication no. 8–23, revised 1985) and the Regulations of the Animal Care and Use Committee of the Experimental Animal Center at Shenzhen Center for Disease Control and Prevention (SZCDCP). This study was approved by the SZCDCP Ethics Committee.

Mice of strain B6129SF2/J (JAX stock #101045) were purchased from Jackson Laboratory (Maine, USA). These F2 hybrid mice are the offspring of an F1 × F1 mating, itself the product of a cross between C57BL/6J females (B6) and 129S1/SvImJ males (129S) [11]. Ten mice were housed in per cage (470 × 350 × 200 mm) with sufficient water and food. The nutritional profile of the diet was as follows: moisture 8.02%, crude protein 22.30%, crude fat 5.35%, crude fiber 4.45%, crude ash 6.36%, calcium 1.332%, total phosphorus 0.673%, copper 20.8 mg/kg, iron 209 mg/kg, magnesium 2182 mg/kg, manganese 91.5 mg/kg, potassium 5317 mg/kg, zinc 111 mg/kg, sodium 2704 mg/kg, selenium 0.424 mg/kg, total arsenic 0.315 mg/kg, chromium 3.08 mg/kg, iodine 2.318 mg/kg, vitamin E 146.1 mg/kg, vitamin A 17670 IU/kg, and vitamin C 9.397 mg/kg, mercury-free and lead-free. Male animals were of two age groups: 4 months old (*n* = 10) and 16 months old (*n* = 10). The animal room has a stable indoor environment: temperature (20 ± 2°C), humidity (55 ± 5%), and 12 hours of light and dark cycle.

### 2.2. Sample Protein Extraction for Proteomic Analysis

After euthanizing mice with 1% sodium pentobarbital, we performed a series of operations to ensure the freshness of the samples, including removing the mouse brain from the skull, excising the hippocampus and cerebral cortex on an ice-cold plate, rapidly freezing tissues in liquid nitrogen, and finally storing the tissue at −80°C. Samples (five or six for each group) were suspended in lysis buffer, containing 8 M urea in PBS, pH 8.0, 1 cocktail, and 1 mM phenylmethanesulfonyl fluoride (PMSF), and ultrasonicated for 90 s (4 s on and 6 s off) at 45% power with a Fisher 550 Sonic Dismembrator (Pittsburgh, PA, USA). After 30-minute incubation time, we proceed as follows: removing debris from the sample centrifugation at 12,000 × g at 4°C for 15 min and transferring the supernatant to a fresh 1.5 mL tube, using Nanodrop 2000c (Thermo Fisher Scientific, Waltham, MA, USA) to determine the protein concentration.

### 2.3. Tandem Mass Tag Labeling

**Figure 1:**
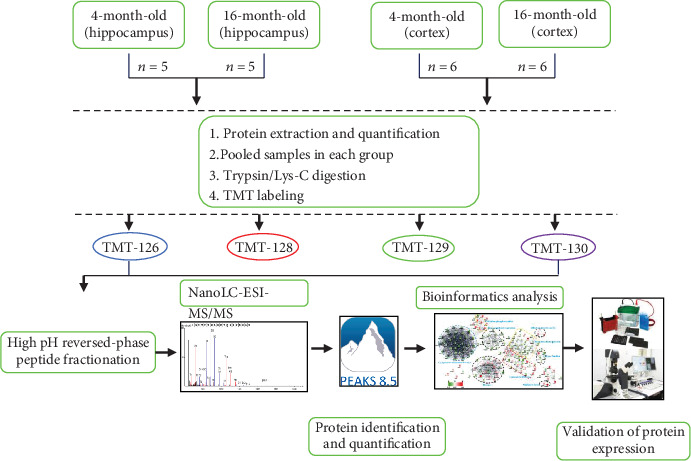
Experimental flowchart. We employed 4-month-old and 16-month-old mice, respectively, each group of 10 mice. From each group, hippocampal and cerebral cortical tissues of 5 or 6 animals were randomly selected for proteomic analysis. For each group, proteins were extracted from 5 or 6 individual samples and pooled (1 : 1 : 1 : 1 : 1 or 1 : 1 : 1 : 1 : 1 : 1) for subsequent proteomic experiments. The pooled proteins were digested into peptides using trypsin/Lys-C Mix and subjected to 4-plex TMT labeling, High pH reversed-phase peptide fractionation and subsequent NanoLC-ESI-MS/MS analysis. The raw data were identified and quantified using PEAKS 8.5 software and subjected to bioinformatics analysis (GO, KEGG, STRING, and Wiki pathways). Selected key proteins were verified by Western blot.

The unilateral hippocampus and cortex of 5 or 6 mice were randomly selected from each group, and 100 *μ*g totaled protein was pooled in equal proportions (1 : 1 : 1 : 1 : 1 or 1 : 1 : 1 : 1 : 1 : 1) for subsequent proteomic analysis (Figure 1). The mixed proteins (per group) were incubated with 10 mM dithiothreitol (DTT, Sigma-Aldrich) at 55°C for 1 h and then incubated with 25 mM iodoacetamide (IAA, Sigma-Aldrich) in the dark at room temperature (RT) for 1 h and digested for 12 h at 37°C with trypsin/Lys-C Mix (Promega, V5072) (protease : protein ratio of 1 : 50). After terminating the reaction, the samples (per group) were acidified with 1% formic acid (FA, Thermo Fisher 167136), desalted with a reverse phase column (Oasis HLB; Waters, MC), and dried with a vacuum concentrator (RVC 2-18 CD plus), then dissolved in TEAB (triethylammonium bicarbonate buffer, 200 mM, pH 1/4 8.5, Thermo Fisher 90114).

According to the TMT 4-plex reagent (Thermo Fisher 90066) instructions, the samples (per group) were labeled with different TMT labels. The 4- and 16-month-old mouse hippocampal/cortical samples were individually labeled with TMT-126, TMT-128, TMT-129, and TMT-130, respectively. Samples (per group) added into the TMT tag were incubated for 1 hour at RT, followed by the addition of 5% hydroxylamine (Thermo Fisher 90115) to terminate the reaction, and finally, the TMT-labeled peptides were mixed together for subsequent experiments.

### 2.4. High-pH Reversed-Phase Chromatography Separation

Labeled peptides were fractionated by Pierce High-pH Reversed-Phase Peptide Fractionation Kit (Thermo Fisher 84868) for further NanoLC-ESI-MS/MS analysis. First, the adjustment of the spin columns consisted of removing the solution, packing the resin material, and washing the spin column twice with 300 *μ*L acetonitrile (ACN) and 0.1% FA solution, respectively, all of which were centrifuged at 5,000 × g for 2 min. Second, peptide separation involved dissolving each sample with 300 *μ*L of 0.1% FA, loading onto a column, and eluting twice with a gradient elution solution of 300 *μ*L (ACN: triethylamine) (Thermo Fisher 84868 (5%, 10%, 12.5%, 15%, 17.5%, 20.0%, 22.5%, 25.0%, and 50.0%)), all of which were maintained at 3,000 × g for 2 min. After drying in a vacuum concentrator, 20 *μ*L of 0.1% FA was added to each tube for NanoLC-ESI-MS/MS analysis.

### 2.5. Data Collection of TMT-Labeled Peptides Using NanoLC-ESI-MS/MS

We loaded the peptide fraction from 2.4 into a ChromXP C18 (3 *μ*m, 120 Å) trap column and analyzed using TripleTOF 5600+ mass spectrometer (SCIEX, Concord, ON, Canada). To acquire ESI/MS/MS data, running in positive ion mode, the first MS scan range is 400-1800 m/z, based on survey scans (250 ms) and acquisition of up to 40 ions in 80 ms (threshold value 160 cps, charge number 2-5) for IDA (information-dependent acquisition), and dynamically excludes selected ions due to the randomness of parent ion selection during MS/MS IDA. In this process, the rolling collision energy setting is adapted to the dissociation of all precursor ions.

Overall, protein extracts from selected brain regions of 5 or 6 mice were mixed before digestion, and then the mixtures were subjected to TMT labeling, peptide fractionation, and NanoLC-ESI-MS/MS analysis (Figure 1). For each group, the proteins were extracted from 5 or 6 individual samples and pooled (1 : 1 : 1 : 1 : 1 or 1 : 1 : 1 : 1 : 1 : 1) for subsequent proteomic analysis. The pooled proteins were digested into peptides using trypsin/Lys-C mix and subjected to 4-plex TMT labeling, high-pH reversed-phase peptide fractionation, and subsequent NanoLC-ESI-MS/MS analysis (Figure 1).

### 2.6. Database Searching and Protein Quantification

We use PEAKS 8.5 software (Bioinformatics Solutions, Waterloo, Canada) to identify and quantify proteins (Figure 1). The UniProt-*Mus musculus* database, containing 51,697 protein entries (released in July 2017), can be used to search the raw mass spectra. The parameters were as follows: parent mass error tolerance of 30 ppm and fragment mass error tolerance of 0.1 Da; FDR (false discovery rate) ≤ 1.0% at peptide-spectrum match level, which can be determined by PEAKS 8.5 searching the related database; peptide scores (−10log *p*) > 19.5, equivalent to a *p* value of ∼1%, were regarded as confidently identified. The TMT 6-plex method was used to relatively quantify peptides and proteins. The summed area, including all reported ion spectra with TMT tags of identified peptides, was taken as the normalized factor across different samples. According to the Peaks Q algorithm, proteins were considered to be significantly different when the ratio ≥ 1.2 or<0.80-fold and the protein significance score ≥ 5.

### 2.7. Bioinformatics Analysis

Visualization of differential proteins by different software and related databases is as follows: DAVID version 6.7 (https://david-d.ncifcrf.gov/) [12, 13] was used to elucidate the biological function of the identified proteins. STRING database version 10.0 (https://string-db.org/) [14, 15] was used to analyze the protein-protein interaction (PPI) network. For logistic analysis of tissue proteomes, Venn Diagram Generator (http://www.pangloss.com/seidel/Protocols/venn.cgi) was used. To analyze Wiki paths, Cytoscape 3.6.1 software and plug-ins were used to STRING-generated network and Wiki path visualization analysis [16, 17].

### 2.8. Western Blot Analysis

After protein quantification, we added the loading buffer for protein denaturation. Then, the samples are separated on SDS-PAGE, transferred to PVDF membranes, and blocked with 5% skim milk. After treatment with the primary antibody (Table 1) of the target proteins and the corresponding secondary antibody, we performed an exposure development operation using an ECL kit (Thermo Scientific Pierce ECL, USA) and analyzed using Image Quant 1D software (GE Healthcare, Pittsburgh, PA, United States).

### 2.9. Assay of ATP Content

ATP levels were determined with an ATP Assay Kit (S0026, Beyotime Institute of Biotechnology, Haimen, China). Approximately 200 *μ*L of lysate was added per 20 mg of tissue, and the mixture was homogenized with a glass or other homogenizer to affect complete tissue lysis. Lysed cells were centrifuged at 12, 000 × g for 5 min at 4°C, and the supernatant was taken for subsequent measurement. We can calculate the relative ATP level with the ratio of the ATP value to the protein value. These values were measured with a multifunctional microplate reader (Tecan Infinite M1000 PRO, Männedorf, Switzerland).

### 2.10. Immunofluorescence Staining

We performed immunofluorescence staining as described previously [18]. Briefly, coronal mouse brain slices were cut into 30 *μ*m thick sections and rinsed 4 times with PBS for 5 min. For immunochemistry of FIS1 and 8-hydroxydeoxyguanosine (8-OHdG), sections were incubated at 4°C for 12 h with the primary antibody (FIS1 at 1 : 100, 8-OHdG at 1 : 100) (Table 1), then washed with PBST and stained with the secondary antibody (1 : 200) for 1 h, Alexa Fluor ®-488 goat anti-rabbit, and Alexa Fluor ®-488 donkey anti-goat (Invitrogen, USA), counterstained for 5 min with DAPI (Beyotime, Haimen, China) to reveal the nuclei, and analyzed using a laser scanning confocal microscope (Leica, Wetzlar, Germany).

### 2.11. Statistical Analysis

The data were expressed as the mean ± SEM with GraphPad Prism 7.0 (GraphPad Software, Inc.), assessed with Student's *t*-test about the level of significance between two groups, and *p* value < 0.05 was considered to be significant.

## 3. Results

### 3.1. Differentially Expressed Hippocampal and Cortical Proteins

**Figure 2:**
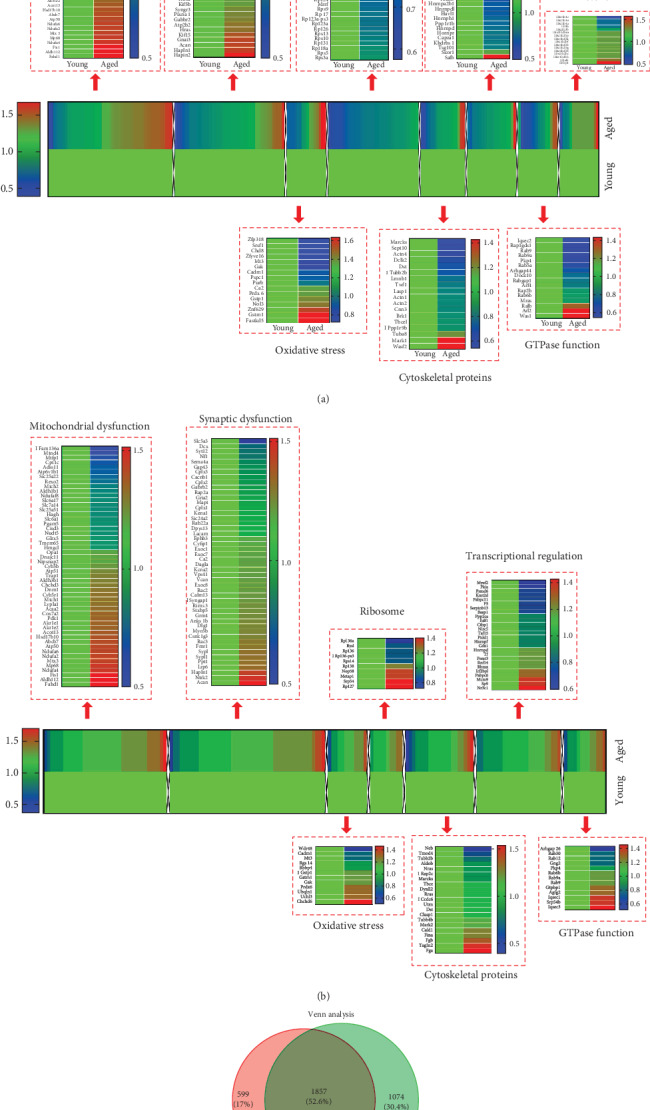
Differentially expressed hippocampal and cortical proteins in aged vs. young mice identified by NanoLC-ESI-MS/MS. (a and b) Proteins differentially expressed in 390 hippocampi and 258 cerebral cortices were hierarchically clustered for 4-month and 16-month-old mice. Differential expression was defined as at least 1.2 times (increased) or ≤0.8-fold (decreased) expression in aged vs. young animal brain. The color of each cell represents the expression level of the protein: red signifies an increase and blue a decreased level relative to that of the control group (4-month-old mice). *n* = 5/6 for per group. Identified proteins are primarily involved in mitochondrial dysfunction, synaptic dysfunction, oxidative stress, ribosome, cytoskeletal integrity, transcriptional regulation, and GTPase function. (c) The Venn logic diagram between the dysregulated proteins of the hippocampus and cerebral cortex in 4-month and 16-month-old mice.

All the proteins of the hippocampus and cerebral cortex were identified by the mass spectra (Supplementary Excel 1). By using the LC-MS/MS analysis combined with TMT labeling technology, based on two unique peptides, we identified a total of 3530 proteins in the hippocampus and cerebral cortex with a false discovery rate (FDR) of less than 1% (Figure 2(c)). Among these proteins, a total of 1857 were common to the hippocampus and cortex (Figure 2(c)). Proteins with at least 1.2 or <0.80-fold and significant score ≥ 5 were considered differentially expressed. We can get the names, accession numbers, and relative abundance ratios of the differential proteins Figures 2(a) and 2(b) from the Swiss-Prot database.

### 3.2. Aging Contributes to Proteomic Alterations in a Mouse Hippocampus

**Figure 3:**
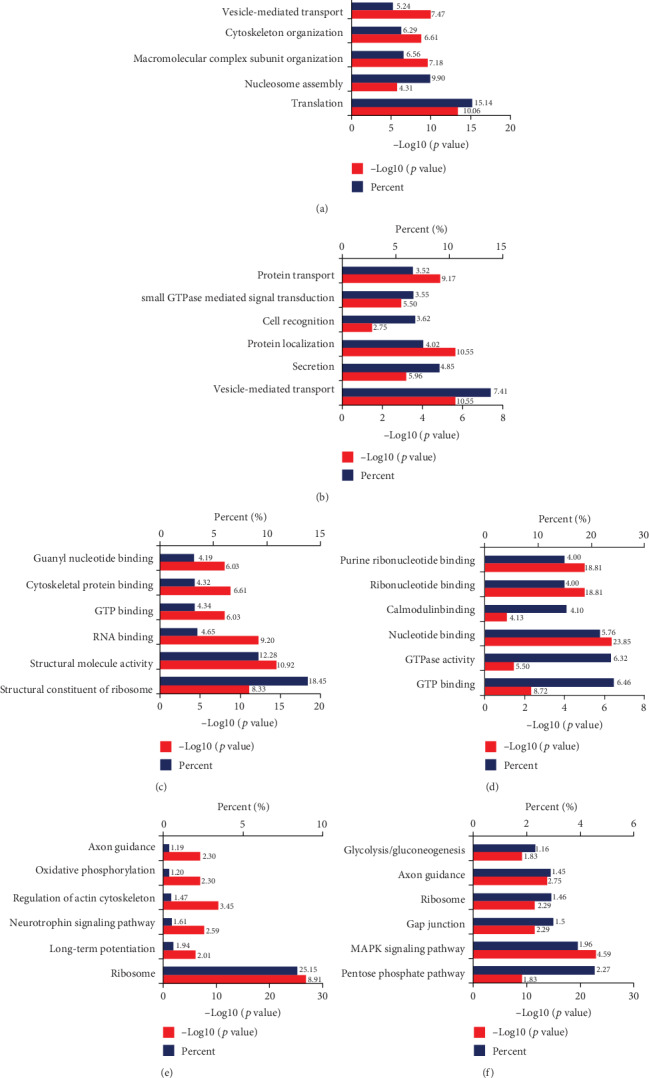
DAVID Gene Ontology and KEGG enrichment analysis for the hippocampal and cerebral cortical proteins of young vs. aged mice. Enrichment analysis for the following: (a) hippocampal proteomics by biological processes, (b) cerebral cortical proteomics by biological process, (c) hippocampal proteomics by molecular function, (d) cerebral cortical proteomics by molecular function, and (e and f) hippocampal and cerebral cortical proteomics by KEGG analysis, respectively.

Three hundred-ninety hippocampal proteins were differentially expressed between 4- and 16-month-old mice (Figure 2(a)). Among these proteins, 121 proteins were increased and 269 decreased in aged vs. young mice; these involved mitochondria (29 increased and 22 decreased), synapses (12 increased and 33 decreased), oxidative stress (7 increased and 9 decreased), cytoskeletal integrity (3 increased and 15 decreased), ribosome (37 decreased), transcriptional regulation (2 increased and 18 decreased), GTPase function (3 increased and 13 decreased), and histone (11 increased and 5 decreased). Gene ontology analysis was performed to reveal the strongly enriched biological processes: translation process, nucleosome assembly process, macromolecular complex subunit organization process, vesicle-mediated transport process, cytoskeleton organization process, and regulation of synaptic transmission process (Figure 3(a)). In addition, we also revealed the strongly enriched molecular function of differential proteins: structural constituent of ribosome activity, structural molecule activity, RNA binding activity, GTP binding activity, cytoskeletal protein binding activity, and guanyl nucleotide binding activity (Figure 3(c)). Further KEGG analysis indicates that these differential proteins are also highly enriched in some pathways associated with aging: ribosome, long-term potentiation, neurotrophin signaling pathway, regulation of actin cytoskeleton, oxidative phosphorylation, and axon guidance (Figure 3(e)).

### 3.3. Aging Contributes to Proteomic Alterations in a Mouse Cerebral Cortex

Two hundred-fifty-eight cortical proteins were differentially expressed between 4- and 16-month-old mice (Figure 2(b)). Among these proteins, 149 proteins were increased and 109 decreased in aging vs. young mice, involving mitochondria (25 increased and 12 decreased), synapses (29 increased and 19 decreased), oxidative stress (8 increased and 5 decreased), cytoskeletal integrity (7 increased and 14 decreased), ribosome (5 increased and 5 decreased), transcriptional regulation (10 increased and 16 decreased), and GTPase function (8 increased and 5 decreased).

Gene ontology analysis was performed to reveal the strongly enriched biological processes: vesicle-mediated transport process, secretion process, protein localization process, cell recognition process, small GTPase-mediated signal transduction process, and protein transport process (Figure 3(b)). In addition, we also revealed the strongly enriched molecular function of differential proteins: GTP binding activity, GTPase activity, nucleotide binding activity, calmodulin binding activity, ribonucleotide binding activity, and purine ribonucleotide binding activity (Figure 3(d)). Further KEGG analysis indicates that these differential proteins are also highly enriched in some pathways associated with aging: pentose phosphate pathway, MAPK signaling pathway, gap junction, ribosome, axon guidance, and glycolysis (Figure 3(f)).

### 3.4. Bioinformatics Analysis for Hippocampal/Cortical Proteins

**Figure 4:**
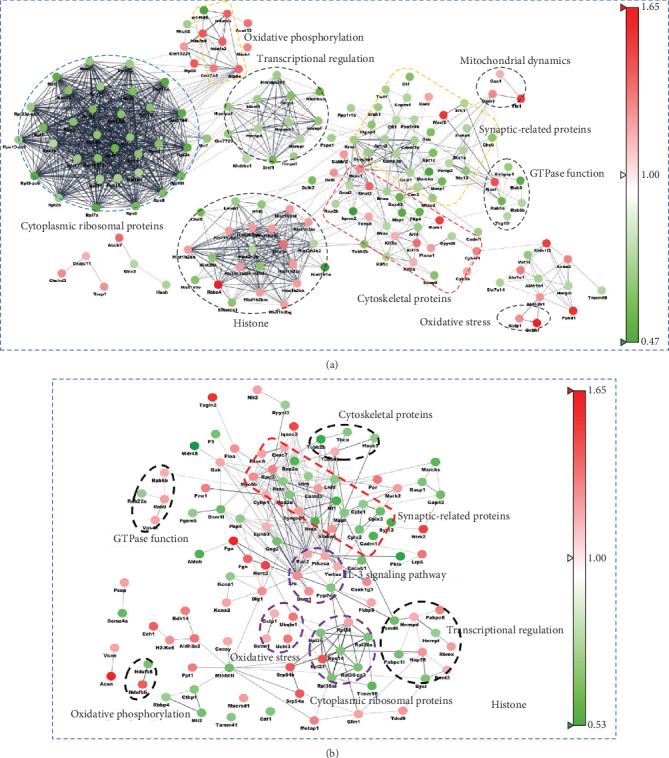
Protein-protein interaction analysis of 390 hippocampal proteins and 258 cortical proteins using the STRING database. All the differentially expressed proteins were visualized and mapped using Cytoscape 3.6.1. STRING analyses of hippocampal differentially expressed proteins (a) and cerebral cortical differential proteins (b). Unconnected proteins were removed from the networks. Interactions between two proteins are indicated with gray edges. Red and green nodes indicate increased or decreased protein levels, respectively.

To identify the potential relationships among the proteins, as shown in Figures 4(a) and 4(b), we used Cytoscape 3.6.1 to visualize the STRING network. We found that many proteins in the PPI map are associated with brain aging processes: cytoplasmic ribosomal pathway, transcriptional regulation, synapses, oxidative phosphorylation, mitochondrial dynamics, oxidative stress, GTPase function, and IL-3 signaling pathway were associated with the aged brain. This indicated that brain aging may involve the deterioration of multiple cellular pathways including the cytoplasmic ribosomal pathway, transcriptional regulation, synapses, mitochondrial dysregulation, and oxidative stress.

**Figure 5:**
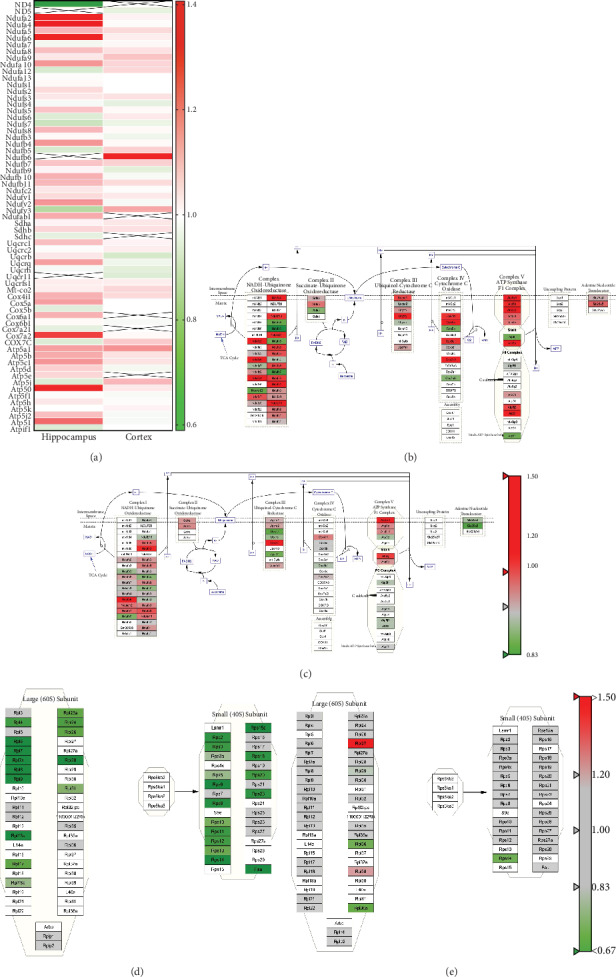
Wiki pathway analysis of all proteins. (a) Heat map analysis of hippocampal and cerebral cortical electron transport chain proteins. Red and green indicate increased or decreased protein levels in aged vs. young mouse brain, respectively. X indicates unscreened protein. Electron transport chain in the hippocampus (b) and cerebral cortex (c) and cytoplasmic ribosomal proteins in the hippocampus (d) and cerebral cortex (e). All proteins were mapped to the related Wiki pathway based on the published database. Proteins are represented by boxes labeled with the protein's name. Relative protein levels in 16-month-old mouse brain compared to those in 4-month-old animals are indicated by colors. Red and green indicated increased or decreased levels, respectively. Proteins in white were not identified in this study.

Based on the PPI map, aging had a greater impact on the hippocampus, which leads to collective imbalances in multiple hippocampal features including transcription, ribosomes, and synapses (Figure 4(a)). Interactions among the hippocampal proteins related to ribosomal metabolism were evident, including the following: 60S ribosomal protein L4 (RPL4), 60S ribosomal protein L7 (RPL7), 60S ribosomal protein L6 (RPL6), 60S ribosomal protein L7a (RPL7a), 60S ribosomal protein L10 (RPL10), 60S ribosomal protein L34 (RPL34), 60S ribosomal protein L8 (RPL8), 60S ribosomal protein L9 (RPL9), 60S ribosomal protein L28 (RPL28), 60S ribosomal protein L13a (RPL23a), 40S ribosomal protein S3 (RPS3), 40S ribosomal protein S15a (RPS15a), 40S ribosomal protein S30 (RPS30), and 40S ribosomal protein S14 (RPS14) (Figure 5(d)). Furthermore, Wiki pathway analysis revealed that cytoplasmic ribosomal proteins were generally decreased in the aged hippocampus, with only a few disorders in the corresponding cerebral cortex (Figure 5(e)). Collectively, the translation function of hippocampal protein in aged mice was degraded.

Proteomics analysis revealed an increase in a large number of electron transfer- (ECT-) related proteins; such changes in the hippocampus were more pronounced in the cerebral cortex (Figure 5(a)). Wiki pathway analysis also revealed that these dysregulated proteins are located in the electron transport chain Complexes I-V (Figures 5(b) and 5(c)) such as Complex I subunits (NDUAA, NDUA2, NDUA4, and NDUA6), Complex II subunits (SDHB), Complex III subunits (QCRI, QCR8), Complex IV subunits (COX5A, COX5B), and Complex V subunits (ATP5A, ATP5H). Collectively, these suggest a disordered electron transport chain in the aged brain.

In sum, proteomic alterations detected in the aged vs. younger brain revealed a large number of protein disorders, particularly impacting hippocampal transcription, translation, and synaptic and mitochondrial proteins.

### 3.5. Further Validation of the Dysregulated Expressed Proteins

**Figure 6:**
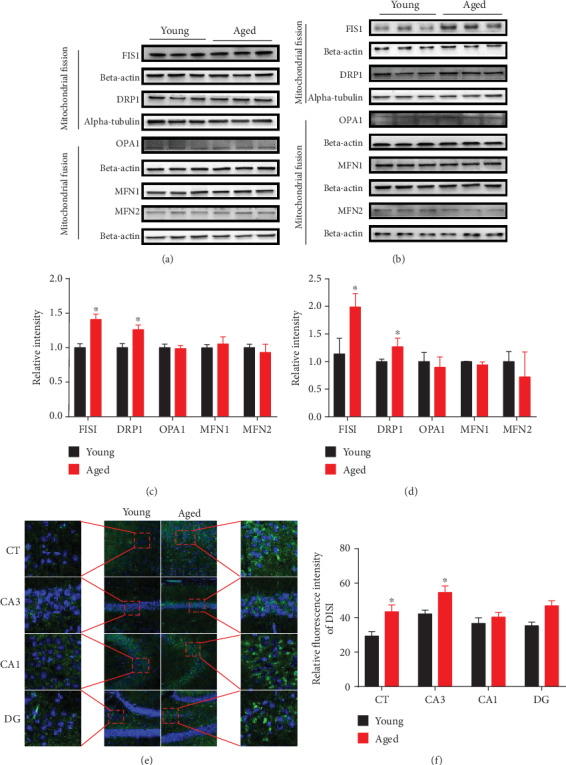
Validation of differentially expressed hippocampal and cortical mitochondrial dynamics proteins. (a and c) Relative levels of hippocampal mitochondrial dynamic proteins in 16-month-old mice compared to 4-month-old mice; (b and d) Relative levels of cerebral cortical mitochondrial dynamics proteins in aged vs. young animals. Data are presented as the mean ± SEM. ^∗^*p* < 0.05 vs. the control mice. *n* = 3 for each group. (e and f) The brain sections containing hippocampal CA1, CA3, dentate gyrus (DG), and cerebral cortex (CT) were stained with anti-FIS1 antibody to detect the level of mitochondrial fission protein. The representative images were selected from 4-month-old mice and 16-month-old mice. Scale bar = 100 *μ*m. Data are presented as the mean ± SEM. ^∗^*p* < 0.05 vs. the control mice.

**Figure 7:**
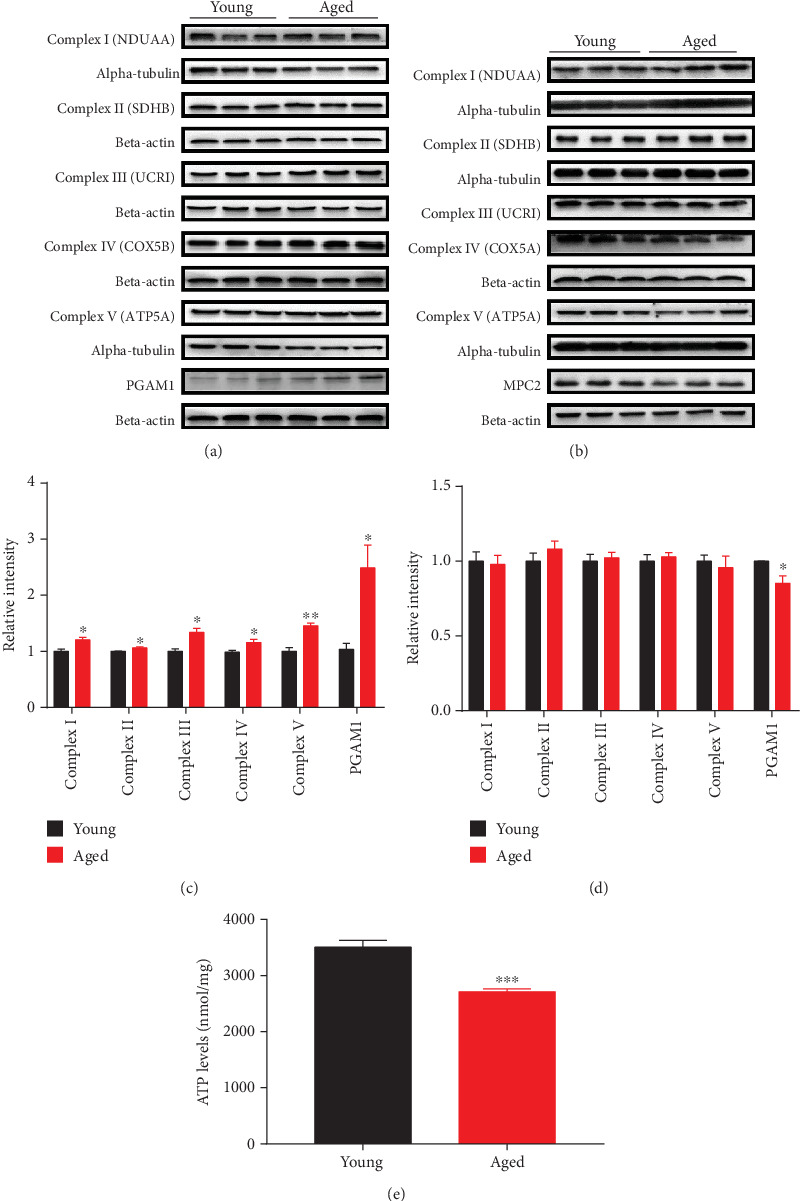
Validation of differentially expressed energy metabolism proteins by Western blot analysis. Relative levels of hippocampal energy metabolism proteins (a and c) and cerebral cortical energy metabolism proteins (b and d) in 16-month-old mice compared to 4-month-old mice. Data are presented as the mean ± SEM. ^∗^*p* < 0.05 vs. the control mice. *n* = 3 for each group. (e) ATP levels in the hippocampus of 4-month-old mice and 16-month-old mice. ATP levels in young and aged animals were determined with an ATP Assay Kit. Data are presented as the mean ± SEM. ^∗^*p* < 0.05 vs. the control mice. *n* = 3 for each group.

**Figure 8:**
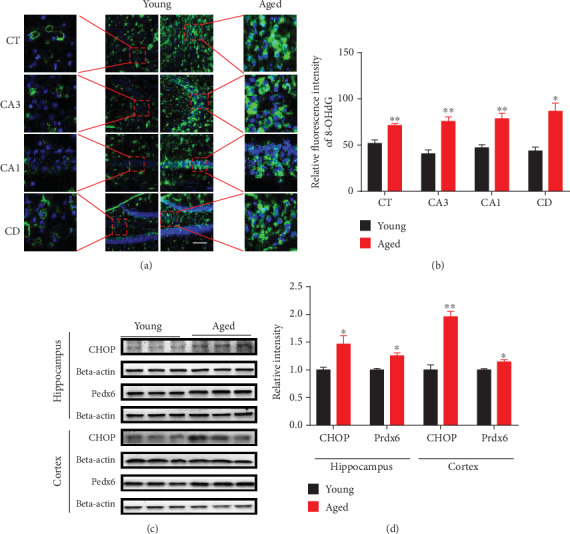
Elevation of PRDX6, CHOP, and 8-OHdG in the hippocampus and cerebral cortex of aged vs. young mice. (a and b) Brain sections containing hippocampal CA1, CA3, dentate gyrus (DG), and cerebral cortex (CT) were stained with anti-8-OHdG antibody to detect the level of these DNA lesions. Representative images were selected from 4-month-old mice and 16-month-old mice. Scale bar = 100 *μ*m. Data are presented as the mean ± SEM. ^∗^*p* < 0.05 and ^∗∗^*p* < 0.01 vs. the control mice. (c and d) The levels of PRDX6 and CHOP were measured by Western blot analysis. Data are shown as the mean ± SEM. ^∗^*p* < 0.05 vs. the control mice. *n* = 3 for each group.

**Figure 9:**
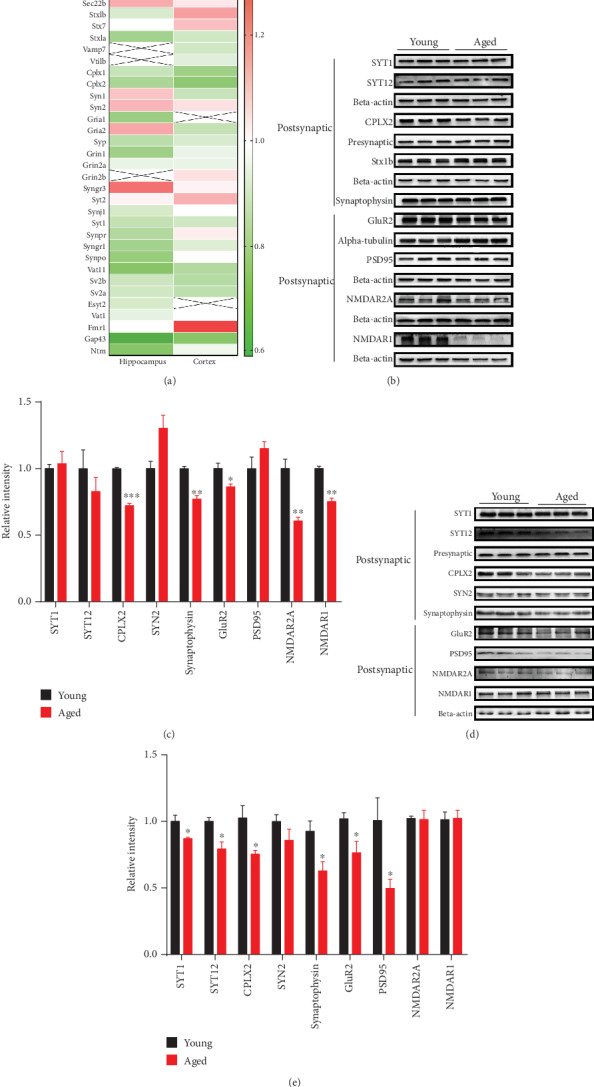
Validation of differentially expressed synaptic proteins by Western blot analysis. (a) Heat map analysis of synaptic proteins in the hippocampus and cerebral cortex. Red and green, respectively, indicate increased or decreased levels in 16-month-old vs. 4-month-old mice. X indicates unscreened protein. (b and c) Relative levels of hippocampal synaptic proteins in aged vs. young mice. (d and e) Relative levels of cerebral cortical synaptic proteins in aged vs. young mice. Data are presented as the mean ± SEM. ^∗^*p* < 0.05, ^∗∗^*p* < 0.01, and ^∗∗∗^*p* < 0.001 vs. the control mice. *n* = 3 for each group.

Brain proteomic analysis revealed abnormal expression of mitochondrial dynamics, oxidative stress, synapse, energy metabolism, and ribosomal metabolism-related proteins in the hippocampus and cerebral cortex of aged vs. young mice. For mitochondrial dynamics, we determined the expression of key molecules involved in mitochondrial fission by Western blot and immunofluorescence staining analysis (Figures 6(a)–6(f)). For energy metabolism, we examined the expression of subunits involved in the electron transport chain and the glycolysis-related protein phosphoglycerate mutase 1 (PGAM1) and by Western blot analysis (Figures 7(a)–7(d)). We also validated oxidative stress-related protein Peroxiredoxin 6 (PRDX6) (Figures 8(c) and 8(d)). Brain proteomic analysis also revealed a relative decrease of many synapse-related proteins: of these, some representative proteins, such as Syntaxin12 (STX12), complexin-2 (CPLX2), synapsin-2, glutamate receptor 2 (GLUR2), synaptophysin, PSD95, NMDAR2A, and NMDAR1 were confirmed by Western blot analysis (Figures 9(a)–9(e)). Consistent with the proteomics data, glycolysis-related protein PGAM1 and synaptic proteins were decreased significantly, while ECT-related proteins, mitochondrial fission protein FIS1, and oxidative stress protein PRDX6 were increased significantly in aged vs. young mice. All verification raw data of Western blot analysis are shown in Supplementary Excel 2.

### 3.6. Decreased ATP Levels in the Hippocampus of Aged in Comparison with Young Mice

To further investigate the effects of aging on energy metabolism, we evaluated ATP levels in the hippocampus of aged vs. young mice. The former showed significantly reduced ATP levels (Figure 7(e)), suggesting that aging is linked with mitochondrial dysfunction in the murine hippocampal respiratory chain.

### 3.7. DNA Oxidative Damage in Aged Mice

To determine the potential effects of aging on regional brain DNA, we measured two markers of DNA damage, namely, CHOP and 8-OHdG. Quantification of green fluorescence intensity showed that 8-OHdG immunoreactivity was markedly increased in the cerebral cortex and hippocampal regions CA1, CA3, and DG of aged vs. young mice (Figures 8(a) and 8(b)). Western blot analysis showed that CHOP was also significantly increased in the cerebral cortex and hippocampus of aged mice vs. young mice (Figures 8(c) and 8(d)). These data suggest that brain DNA oxidative damage accrues with aging.

### 3.8. Decreased BDNF Levels in the Hippocampus of Aged Mice

**Figure 10:**
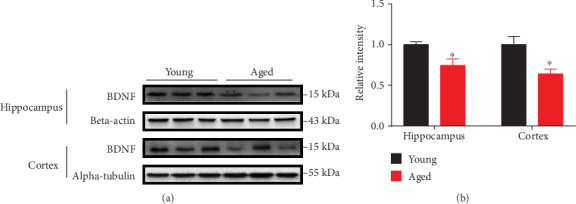
Expression level of BDNF analyzed by Western blot. (a and b) Relative levels of hippocampal and cortical BDNF in 16-month-old mice compared to 4-month-old mice. Data are shown as the mean ± SEM. ^∗^*p* < 0.05 vs. the control mice. *n* = 3 for each group.

Considering the broad role of BDNF in neuroprotection, including synaptic plasticity, oxidative, metabolic, and excitotoxic stress [19, 20], we explored the expression changes of BDNF in the hippocampus and cerebral cortex of aged vs. young mice. Western blot analysis showed significantly reduced levels of BDNF in both the cerebral cortex and hippocampus of aged mice vs. young mice (Figures 10(a) and 10(b)).

## 4. Discussion

Proteomic analysis detected a total of 3530 proteins, of which 1857 proteins were common to the hippocampus and cerebral cortex. 390 hippocampal proteins and 258 cerebral cortical proteins showed altered levels in the 16-month-old vs. the 4-month-old mouse brain, and these proteins were involved in mitochondrial function, energy metabolism, synaptic function, the cytoplasmic ribosomal pathway, transcriptional regulation, and oxidative stress. Aging seemed to have a more pronounced effect on protein levels in the hippocampus than those in the cerebral cortex, as noted in previous proteomic studies of mouse brain [10, 21], whereas the main pathways affected are shared between the two regions encompassing altered mitochondrial dynamics, synapse proteins, and oxidative stress in the hippocampus and cerebral cortex, with transcriptional regulation, energy metabolism, and changes in the cytoplasmic ribosome pathway more obvious in the hippocampus of aged mice. In this study, samples in each age group were pooled together to evaluate the differences of protein profiles among groups. However, it was difficult to reveal the individual variation in the same group, which is also one of the limitations for this study.

### 4.1. Mitochondrial Dynamics

Our results found aging was associated with simultaneous increase of fission 1 protein (mitochondrial outer membrane) in both the hippocampus and cerebral cortex of aged mice. In addition, dynamin-like protein DRP1 (dynamin 1-like) was significantly increased in the cerebral cortex of aged mice in comparison to young mice. In the pathological study of Alzheimer's disease and Huntington's disease, fis1 was found to aggravate the aggregation of DRP1 in mitochondria by interacting with DRP1 oligomers, which can induce mitochondrial division [22–25]. In addition, FIS1 knockdown promotes mitochondrial fusion and inhibits apoptosis [26, 27]. Compared with nonneuronal cells, DRP1 plays a significant role not only in the regulation of mitochondrial morphology but also in the distribution of mitochondria in axons, dendrites, and synapses [28–33]. Mitochondrial fission is vital for mitotic segregation of mitochondria to daughter cells, distribution of mitochondria to subcellular locations, and mitophagy [34, 35]. Unopposed fission leads to mitochondrial fragmentation, loss of OXPHOS function, mtDNA depletion, and ROS production, which are associated with metabolic dysfunction or disease [36]. Mitochondrial fission may enhance apoptosis by increasing the availability of outer membrane surface area for pore formation. Increased mitochondrial fission proteins disrupt mitochondrial membrane potential and respiration, resulting in slow cell growth and accelerated cell aging [37]. Taken in concert, these findings suggest that aging may dysregulate brain mitochondrial dynamics via increased levels of FIS1 and DRP1.

### 4.2. Energy Metabolism

Brain aging has been previously associated with abnormal energy metabolism, including increases in enzymes associated with glycogen metabolism and altered levels of proteins in the ETC, the function of which is required for ATP production [14, 21, 38]. We found major changes in the levels of energy metabolism-related proteins in the hippocampus and cerebral cortex of aged vs. young mice, and ETC-related proteins were most obviously increased in the hippocampus (Figure 5(a)). As an essential glycolytic enzyme [39], phosphoglycerate mutase 1 (PGAM1) was increased in the hippocampus of aged compared to young mice. However, mitochondrial pyruvate carrier 2 (MPC2), a heterooligomer complex, can be formed in the mitochondrial inner membrane (IMM), involved in pyruvate transport, for mitochondrial pyruvate oxidation and carboxylation [40], decreased in the cerebral cortex of aged mice. Wiki pathway analysis showed a general disorder of ETC-related proteins, Complex I-V subunits [41] (Figures 5(b) and 5(c)). In summary, the abnormal changes in many energy metabolism pathways, including the glycolysis (PGAM1), pyruvate oxidation and carboxylation (MPC2), and oxidative phosphorylation (OXPHOS) [42, 43], suggest that widespread disruption of brain energy metabolism occurs during mouse aging. As a process of the mitochondrial electron transport chain, oxidative phosphorylation is particularly important in the process of producing ATP, but as age increases, electrons leak and increase in reactive oxygen species, causing mitochondrial dysfunction and increased oxidative stress [44].

### 4.3. Oxidative Stress

Concomitantly, glutathione metabolism represents a key defense system that provides resistance to oxidative stress which involves increased protein glutathione S-transferase (GST), peroxidase (PRDX), and superoxide dismutase (SOD). During oxidative stress, GST protects against reactive molecules via catalyzing the nucleophilic attack of glutathione on electrophilic substrates [45]. Furthermore, the H2O2-scavenging peroxiredoxins (Prxs), a thiol-specific antioxidant enzyme with six isozymes (PRDX1, 2, 3, 4, 5, and 6) [46], play a significant effect on the regulation of reactive oxygen species (ROS) [47]. We found increased levels of GSTP1, GSTM1, and PRDX6 in the hippocampus and cerebral cortex of aged mice compared with young animals. A previous study also reported that the protein GSTP1 participating in the regulation of oxidative stress was significantly increased in the brain of aged rats [48]. Recent transcriptomics studies are in accordance with our results, since GST, subunits mu1, mu3, mu7, pi2, theta 1, and theta2 are all significantly increased in aged mouse brain tissue [49]. The increased levels of glutathione metabolism-related proteins GSTP1, GSTM1, and PRDX6 in aged vs. young brain suggest that a protective antioxidant compensatory mechanism becomes more active as aging advances [50].

### 4.4. Synaptic Dysfunction

Synaptic decline and plasticity deficiency contribute to age-related retardation in learning and memory [51], and there is also evidence of deficits in synaptic transmission [52] consistent with the decreased number of synaptic connections with the advance of age [53]. Our proteomics results show that aging promotes an overall imbalance of synapse-associated proteins. Consistent with the proteomics results, levels of complexin-2, synaptophysin, GLUR2, PSD95, NMDAR2A, and NMDAR1 were reduced significantly in aged mice contrasted with young mice. Synaptotagmin-12 is a synaptic vesicle phosphoprotein that regulates the release of spontaneous neurotransmitters, controlled by cAMP-dependent phosphorylation. It is differentially expressed in the aging hippocampus and cerebral cortex of aging mice [54]. Complexin-2 plays an important regulatory role in synaptic structure and can be replaced by synaptic-binding proteins, facilitating transport of vesicles to synaptic membrane [55, 56]. Our observations suggest that aging causes a deficiency of synaptic connections and plasticity, supporting the theory of regulation or diminishment synaptic transmission with aging. A recent proteomics study of mouse brain found that in the learning and memory formation, the expression of some receptors and signaling cascade proteins of young and middle-aged mice is different significantly [10].

### 4.5. Cytoplasmic Ribosomes

Translation is the most important process in the regulation of protein expression that affects the ability of cellular proteins to stabilize, a function associated with the lifespan of multiple model organisms [57]. Numerous studies have shown that many ribosome-associated proteins and translational regulators are closely related to lifespan [58–60]. We found in aged vs. young mice that a large number of cytoplasmic ribosomes-related proteins were decreased in the hippocampus but not significantly altered in the cerebral cortex. In addition, it is well known that translation of ribosomal protein L4 (RPL4) is necessary for rapid axonal regeneration [61] and ribosomal protein S3 (RPS3) is involved in DNA repair mechanisms [62]. In sum, the overall decrease of ribosomal proteins may be closely related to the physiology of brain aging.

### 4.6. Loss of Neurotrophic Factor

BDNF (neurotrophin brain-derived neurotrophic factor) plays an important part in modulating the synaptic function of neurons. Following biosynthesis, the combination of BDNF to TrkB is required to transport BDNF from dense core vesicles to axon terminals, where it is secreted into the synaptic cleft following membrane depolarization [63, 64]. The efficacy of excitatory synapses is enhanced by recombinant BDNF delivery to hippocampal slices [65, 66]. These effects are primarily mediated through changes in NMDA receptor function [67]. A more recent study showed that BDNF can enhance mitochondrial ATP production by increasing respiratory coupling, thus contributing to neuroprotective mechanisms associated with neural plasticity [68].

In addition to neural plasticity, studies have clearly shown that BDNF plays an important role in a variety of nerve damages, and its reduction is closely related to a variety of neurodegenerative diseases. [19, 20]. Moderate amounts of BDNF exposed to hippocampus or cortical neurons prevent acute and/or chronic neurodegenerative diseases associated with: mitochondria toxins [68], oxidative stressors [69], glucose and oxygen deprivation [70], glutamate and excitotoxins [71, 72], and amyloid-*β* peptide [73]. In this study, we found that compared with young mice, the level of BDNF in hippocampus of aged mice was significantly reduced, indicating that decreased BDNF may contribute to brain aging by disrupting multiple complex physiological processes, including mitochondrial dysregulation, energy metabolism, synaptic dysfunction, and oxidative stress.

## 5. Conclusions

In summary, a lot of proteins in the hippocampus and cerebral cortex of aged mice were differentially expressed. Such changes predictably promote the decline of physiological brain function by decreasing ATP content, increasing DNA oxidative damage, and synaptic dysfunction. Hippocampal and cortical proteomics and bioinformatic analysis revealed that aging is related to abnormal expression of proteins related to mitochondrial dynamics (FIS1, DRP1), energy metabolism (PGAM1, MPC2), oxidative stress (PRDX6, GSTM1, and GSTP1), synapses (SYT12, GLUR2), ribosome (RPL4, RPS3), loss of neurotrophic factor, transcriptional regulation, and GTPase function. Through comprehensive proteomic analysis, we can reasonably interpret the functional significance of altered protein profiles in the brains of early aging mice, as well as the relationships and pathways involved in various physiological/pathological processes.

The critical importance of diet composition and housing conditions in regulating the longevity of mice may limit the generalizability of our observations across laboratories. While every effort was made to standardize these variables, longevity outcomes may vary across laboratories even when the same strain, diet, and husbandry practices are employed. In this study, 10 male mice were housed in a large cage (470 × 350 × 200 mm). I believe this is a limitation of this study, housing one-group mice in a big cage, although previous data showed no significant change in the aging characteristics explored between the mice (8-10 mice) housed in a big cage and the mice housed in a small age (3-5 mice) [16, 74]. Median lifespan variations of up to 31% (704-925 days) have been observed for a single mouse strain housed and fed under identical conditions in collaborating laboratories, and strain and sex differences can modify responses to dietary restriction, which is known to extend the lifespan. Furthermore, the food intake of mice, which in turn can affect lifespan, is greater when animals are held in rooms at temperatures lower than their thermoneutral zones (30-34°C) [75].

## Figures and Tables

**Table 1:** Brands and usages of the primary antibodies.

Antibody	Specificity	Type	Dilution	Source	CAT no.
*β*-Actin	Beta-actin	Mouse	1/3000	Santa Cruz	sc-47778
*α*-Tubulin	Alpha-tubulin	Mouse	1/3000	Santa Cruz	sc-73242
FIS1	Fission 1	Rabbit	1/1000	Proteintech	10956-1-AP
DRP1	Dynamin-related protein 1	Mouse	1/1000	Santa Cruz	sc-271583
OPA1	OPA1 (D-9)	Mouse	1/1000	Santa Cruz	sc-393296
MFN1	Mitofusin 1	Mouse	1/1000	Abcam	ab57602
NFN2	Mitofusin 2	Rabbit	1/1000	Proteintech	12186-1-AP
NDUAA	NDUFA10	Rabbit	1/1000	Abcam	ab103026
SDHB	Succinate dehydrogenase subunit B	Mouse	1/1000	Abcam	ab14714
UCRI	UQCRFS1	Rabbit	1/1000	Abcam	ab131152
COX5A	COX5a (A-5)	Mouse	1/3000	Santa Cruz	sc-376907
COX5B	Cytochrome c oxidase subunit 5B	Rabbit	1/1000	Abcam	ab180136
ATP5A	ATP synthase F1 subunit alpha	Mouse	1/1000	Abcam	ab14748
PGAM1	Phosphoglycerate mutase 1	Rabbit	1/1000	Abcam	ab184232
MPC2	Mitochondrial pyruvate carrier 2	Rabbit	1/1000	Cell Signaling	#46141
PRDX6	Peroxiredoxin 6	Mouse	1/1000	Abcam	ab16947
CHOP	CHOP (L63F7)	Mouse	1/1000	Cell Signaling	#2895
SYT1	Synaptotagmin-1	Rabbit	1/1000	Abcam	ab131551
SYT12	Synaptotagmin-12	Rabbit	1/1000	Proteintech	55015-1-AP
CPLX2	Complexin-2	Goat	1/1000	Abcam	ab215046
SYN II	Total synapsin-2	Rabbit	1/1000	Abcam	ab76494
Synaptophysin	Total synaptophysin	Rabbit	1/1000	Abcam	ab32127
GluR 2	Total GLUR2	Rabbit	1/1000	Abcam	13607s
PSD-95	Total postsynaptic density 95	Rabbit	1/1000	Abcam	ab76115
NMDAR 2A	Total NMDA receptor 2A	Rabbit	1/1000	Abcam	ab124913
NMDAR 1	Total NMDA receptor 1	Rabbit	1/1000	Abcam	ab109182
BDNF	Brain-derived neurotrophic factor	Rabbit	1/1000	Abcam	ab108319
8-OHdG	8-Oxo-2′-deoxyguanosine	Goat	1/100	Abcam	ab10802

## Data Availability

All data used to support the findings of this study are included within the article. Raw data used to generate the figures are available from the corresponding author upon request.

## Supplementary Materials

Supplementary 1 Supplementary Excel 1: all the proteins of the hippocampus and cerebral cortex identified by the mass spectra.

Supplementary 2 Supplementary Excel 2: all verification raw data of Western blot analysis.
